# Conservation Research Is Not Happening Where It Is Most Needed

**DOI:** 10.1371/journal.pbio.1002413

**Published:** 2016-03-29

**Authors:** Kerrie A. Wilson, Nancy A. Auerbach, Katerina Sam, Ariana G. Magini, Alexander St. L. Moss, Simone D. Langhans, Sugeng Budiharta, Dilva Terzano, Erik Meijaard

**Affiliations:** 1 School of Biological Sciences, The University of Queensland, Brisbane, Queensland, Australia; 2 Institute of Entomology, Biology Centre CAS, České Budějovice, Czech Republic; 3 Faculty of Science, University of South Bohemia in České Budějovice, České Budějovice, Czech Republic; 4 KPMG Botswana, Gaborone, Botswana; 5 Leibniz-Institute of Freshwater Ecology and Inland Fisheries, Berlin, Germany; 6 Indonesian Institute of Sciences, Pasuruan, East Java, Indonesia; 7 School of Geography, Planning and Environmental Management, The University of Queensland, Brisbane, Queensland, Australia; 8 Borneo Futures Initiative, Ciputat, Jakarta, Indonesia

## Abstract

Target 19, set by the Convention on Biological Diversity, seeks to improve the knowledge, science base, and technologies relating to biodiversity. We will fail to achieve this target unless prolific biases in the field of conservation science are addressed. We reveal that comparatively less research is undertaken in the world’s most biodiverse countries, the science conducted in these countries is often not led by researchers based in-country, and these scientists are also underrepresented in important international fora. Mitigating these biases requires wide-ranging solutions: reforming open access publishing policies, enhancing science communication strategies, changing author attribution practices, improving representation in international processes, and strengthening infrastructure and human capacity for research in countries where it is most needed.

## Abstract 2

Conservation science is an applied discipline aiming to improve biodiversity conservation practice, and one might logically expect that most research would therefore be undertaken in the world’s most biodiverse countries. This analysis reveals that it is not.

In the environmental sciences, the scientific process generates evidence for policies and practices. Published evidence indicates that the quality standards associated with peer review have been met. Publishing also provides others with access to the evidence being shared, and increasingly, to the data and methodological processes underlying it. There are, however, strong biases in the peer-reviewed literature.

Biodiversity and the threats to its persistence are not uniformly distributed across the globe and therefore some areas demand comparatively greater scientific attention. If research is biased away from the most biodiverse areas, then this will accentuate the impacts of the global biodiversity crisis and reduce our capacity to protect and manage the natural ecosystems that underpin human well-being. Target 19 of the Convention on Biodiversity (CBD) states that “By 2020, knowledge, the science base, and technologies relating to biodiversity, its values, functioning, status and trends, and the consequences of its loss, are improved, widely shared and transferred, and applied” [1]. Biases in conservation science will prevent us from achieving this target.

We conducted the first comprehensive analysis of publishing trends of the conservation science literature. We identified all publications from 2014 on the topic of “conservation” in the research areas of environmental sciences, ecology, biodiversity conservation, plant sciences, zoology, and geography. We searched both the Thomson Reuters Zoological Records and Web of Science Core Collection databases, which returned 10,036 scientific publications (from 1,061 journals), after the duplicate, unrelated, and incomplete records were removed. For a subset of these publications (*n* = 7,593, or 81%), we manually identified at least one topic country, and we determined the relative conservation importance of these countries for mammal conservation [2] as well as a broader definition of conservation importance that considers richness of vascular plants, endemic species, and functional species [3].

The countries for which knowledge is sparse coincide with where research is most urgently needed. The top five countries, ranked according to relative importance for mammal conservation (i.e, Indonesia, Madagascar, Peru, Mexico, and Australia), were represented in 11.9% of the publications (Table 1A). However, our determination, based on relative importance for investment in mammal conservation, was that these countries should be represented in 37.2% of the publications. We determined that the United States should be represented in approximately 0.5% of the publications—instead, it was the subject of approximately 17.8% of the publications and was the most studied country overall (Fig 1). If we consider the broader definition of conservation importance that reflects the richness of vascular plants, endemic species, and functional species, then the top five countries (i.e., Ecuador, Costa Rica, Panama, the Dominican Republic, and Papua New Guinea) are the focus of only 1.6% of publications (Table 1B). On the basis of the proposed level of investment for mammal conservation alone, we would expect these countries to be represented in at least 7.3% of the publications. Comparatively less research is published on the most biodiverse countries. This situation needs to be redressed before 2020, else we will fail to achieve Target 19. Furthermore, aggregated biodiversity metrics that are used to inform progress against other CBD targets, such as the Living Planet Index and the Red List Index [4,5], will continue to be populated with biased data.

**Fig 1:**
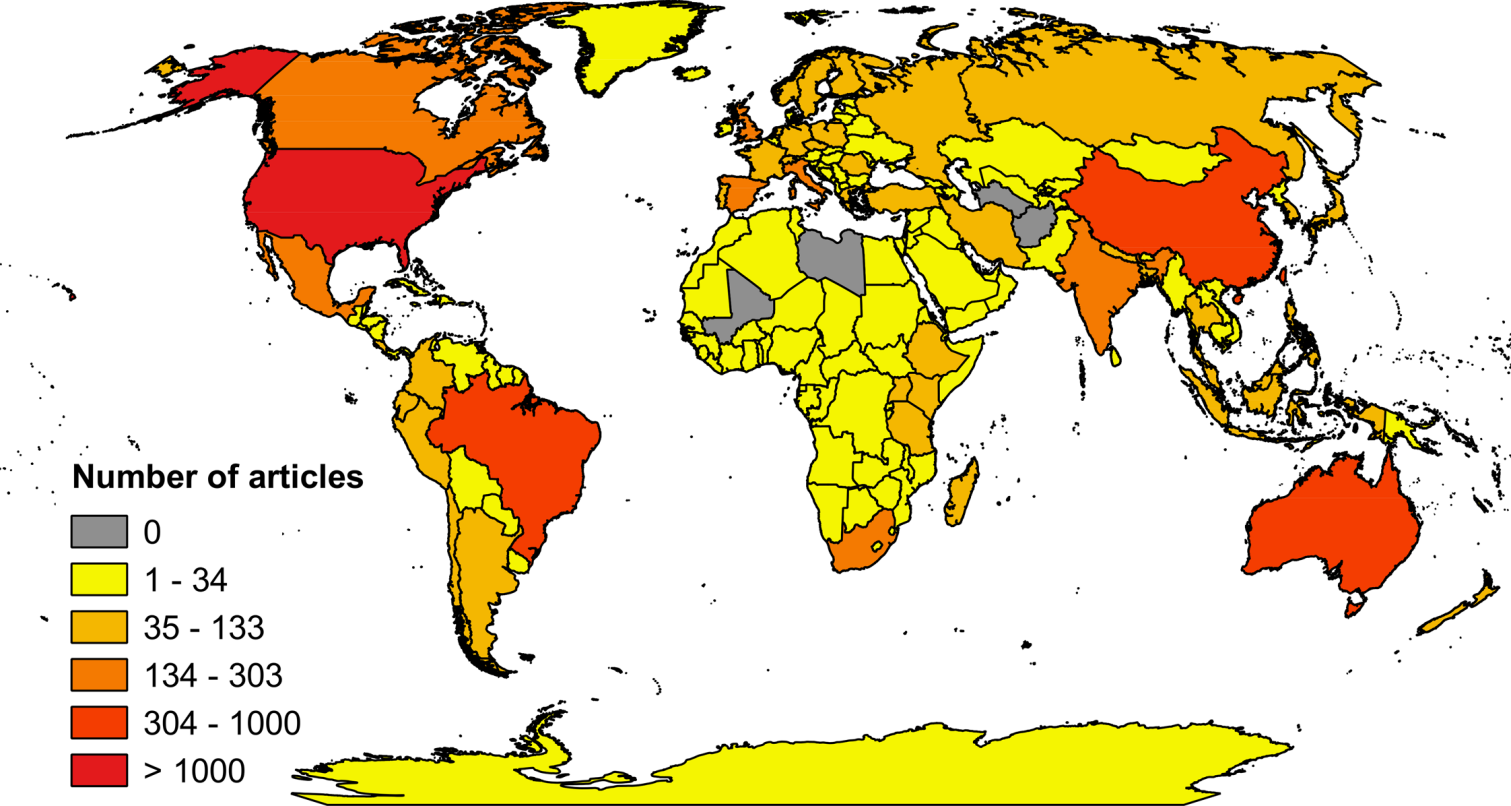
Global distribution of publications on biodiversity conservation (S1 Data).

**Table 1:** Publishing trends and representation in the International Union for Conservation of Nature (IUCN) Specialist Groups or the Intergovernmental Panel on Biodiversity and Ecosystem Services (IPBES) for (A) the countries ranked highest in terms of importance for mammal conservation [2], (B) the countries ranked highest in terms of biodiversity [3], and (C) the United States and United Kingdom, for the purposes of comparison (S1 Data).

Country	Number publications (with % of total)	Percentage publications led by an in-country institution	Average Altmetrics score (with maximum)	Number publications published open access	Number IPBES experts	Number IUCN chairs
**A**						
1. Indonesia	95 (1.1)	23	12.5 (133)	9	5	1
2. Madagascar	64 (0.8)	14	19.8 (194)	7	10	1
3. Peru	49 (0.6)	10	15.2 (105)	11	2	0
4. Mexico	228 (2.8)	68	12.4 (256)	62	9	4
5. Australia	527 (6.5)	94	11.2 (192)	24	21	8
**B**						
1. Ecuador	46 (0.6)	22	9.4 (52)	6	1	0
2. Costa Rica	37 (0.5)	14	3.8 (7)	3	4	0
3. Panama	22 (0.3)	5	3.8 (7)	5	0	0
4. Dominican Republic	6 (0.07)	0	1.5 (2)	0	1	0
5. Papua New Guinea	16 (0.2)	0	9.3 (22)	1	0	0
**C**						
US (ranked 40 for A and 157 for B)	1,441 (17.8)	93	11.8 (434)	71	23	44
UK (ranked 170 for A and 167 for B)	249 (3.1)	77	15 (146)	11	18	39

With comparatively fewer publications being generated, it would be ideal for these publications to be widely shared. Open access publishing is growing in popularity, but still only 14% (*n* = 809) of the publications recorded in the Thomson Reuters Web of Science Core Collection database were published as open access. Only 128 of the 1,090 publications (11.7%) that focused on the ten countries of the greatest conservation importance were freely accessible (Table 1). Publishing open access can incur hefty fees, and the contemporary practice of journals is to waive publishing fees on a case-by-case basis. We encourage parent societies of key conservation journals (e.g., the Society for Conservation Biology and the Ecological Society of America) to openly commit to waiving fees for research from historically underrepresented countries, particularly those where local in-country scientists and institutions have played a significant role in the research.

It is the responsibility of the scientific community to give adequate acknowledgement of in-country scientists and institutions [6], particularly since the research conducted in the most biodiverse countries is predominately led by researchers based elsewhere. Only 23% of the Indonesian publications, 22% of the Ecuadorian, and none of the Papua New Guinean and the Dominican Republic publications were led by researchers affiliated with local institutions (Table 1). This is compared to the publications concerning the US, where 93% were led by authors based at an institution in the US. Of the countries that ranked highly according to our measures of conservation importance, Australia had the greatest rate of in-country publications (94%). Others have observed similar discrepancies, but the extent of the problem was unknown since these studies were focused on specific taxonomic groups, ecosystem services or biomes [7–9], or a limited subset of journals [10,11] or countries [12,13]. Attribution of joint affiliations for lead authors would enable local institutions to be recognised at national levels and by international ranking systems.

While peer-reviewed publications are an important component of evidence-based policy [14], on-ground change necessitates the support of a concerned public [15]. Social media outlets are important mechanisms for widely communicating research findings. Furthermore, engagement in social media contributes to social capital and community participation by creating cohesive networks and enabling the exchange of information across diverse groups [16]. Interestingly, we find evidence that the public is more interested in the research findings from biodiverse countries, as indicated by the Altmetrics score for each publication (a measure of attention generated in social media). The average Altmetrics score for the publications concerning the top five countries for investment in mammal conservation was 14.2 (*n* = 353). A publication concerning the US had the highest score (434), but overall, the publications on the US had a lower average, at 11.8 (*n* = 436) (Table 1C). The impacts of the publishing biases we observe could thus be mitigated by the broad distribution of research through social media, necessitating ongoing investment by the research community in diverse communication strategies.

Target 19 also calls for the knowledge generated to be applied, but researchers from countries of high conservation importance currently have very little representation in international fora. If representation as a chair or co-chair of an International Union for Conservation of Nature (IUCN) Specialist Group (of which there are 182 across 135 groups) is regarded as a measure of impact on conservation practice, then we would conclude that researchers with impact are based predominately in the US (*n* = 44) or the UK (*n* = 39) (Table 1C). Similarly, for the 515 individuals (representing 90 countries) who were selected as experts for the Intergovernmental Panel on Biodiversity and Ecosystem Services (IPBES), the most frequently represented countries are the US (*n* = 23), Brazil (*n* = 25), and Germany (*n* = 25). Of the countries ranked highest according to our measures of conservation importance, only Australia had modest levels of representation in both the IUCN specialist groups (*n* = 8) and IPBES (*n* = 21) (Table 1). We recommend more concerted effort to recruit scientists from the most biodiverse countries in important international fora, which will further enhance knowledge-exchange and the formation of new research collaborations.

Generating research that is of publication quality relies on a significant investment in research infrastructure and human capacity. Surprisingly, we found no correlation between the gross domestic product (GDP) per capita and the number of publications concerning each country. We did, however, find a positive correlation between expenditure on research as a proportion of GDP and the number of publications about a country (*r* = 0.21; *p* < 0.05) and also the proportion of those publications led by a researcher based at a local institution (*r* = 0.35; *p* < 0.05).

Countries where we stand to lose the most biodiversity are currently underrepresented in the peer-reviewed literature and international fora, and this situation will only be reinforced by strong publishing biases. This is a global concern and reduces our capacity to achieve international conservation targets. To mitigate the impacts of these biases, we recommend that the scientific community sponsor open access publications and widely disseminate research results through social media, give greater recognition to and acknowledgment of the contributions of local researchers and institutions, and actively seek to improve the representativeness of international fora. The greatest opportunity to redress publishing bias in conservation science lies in greater in-country investment in research. Ultimately, the onus rests on governments, the private sector, and donors to bolster in-country funding in conservation research where it is most needed.

## Supporting Information

S1 Data (XLSX)

## Abbreviations

CBD: Convention on Biodiversity
IPBES: Intergovernmental Panel on Biodiversity and Ecosystem Services
IUCN: International Union for Conservation of Nature
